# Lack of proportionality. Seven specifications of public interest that override post-approval commercial interests on limited access to clinical data

**DOI:** 10.1186/1745-6215-13-100

**Published:** 2012-07-02

**Authors:** Daniel Strech, Jasper Littmann

**Affiliations:** 1 CELLS – Centre for Ethics and Law in the Life Sciences, Hannover Medical School, Carl-Neuberg Str. 1, 30625, Hannover, Germany; 2 Institute of Biomedical Ethics, University of Zurich, Switzerland; 3 University College London, London, UK

## Abstract

For the protection of commercial interests, licensing bodies such as the EMA and health technology assessment institutions such as NICE restrict full access to unpublished evidence. Their respective policies on data transparency, however, lack a systematic account of (1) what kinds of commercial interests remain relevant after market approval has been granted, (2) what the specific types of public interest are that may override these commercial interests post approval, and, most importantly, (3) what criteria guide the trade-off between public interest and legitimate measures for the protection of commercial interest. Comparing potential commercial interests with seven specifications of relevant public interest reveals the lack of proportionality inherent in the current practices of EMA and NICE.

## Background

Several strategies have been discussed and recommended to avoid or at least decrease the extent of bias in published data [1,2], e.g., a requirement to register studies, greater readiness of academic journals to accept articles that publish negative findings, or commitments of pharmaceutical manufacturers to fully publish all available data. These strategies have not been sufficiently effective. It is well established and has been demonstrated in several recent cases that the publication of clinical trials is often incomplete and that there is an observable bias towards publishing those results that are favourable to a manufacturer of new drugs or medical devices [3-5]. It is also well known, however, that licensing bodies such as the FDA and EMA, which grant market approval for new pharmaceutical products, have access to all unpublished trial data (at least all those that were required to grant market approval) [3,6,7]. Despite repeated calls for more transparency on trial data, the data held by the EMA, for example, are not available for the public. This is also true for national health technology assessment (HTA) institutions such as England's NICE that provide (cost-) effectiveness analyses for reimbursement decisions and will often negotiate the provision of unpublished data with pharmaceutical manufacturers on conditions of confidentiality [8]. The main reasons given by the respective agencies and outlined in the corresponding policy documents to agree to conditions of confidentiality are the protection of the commercial interests of the pharmaceutical or medical device industry (commercial-in-confidence regulations) and the protection of personal data (patient confidentiality) [7-9]. However, the same policy documents also cite a limitation to the protection of commercial interests, namely in cases where commercial interests themselves (or measures for the protection of commercial interests) infringe upon wider public interests. The EMA, for example, specifies, “active publication or disclosure upon request for access to documents can be done when an overriding public interest in disclosure can be identified” [7].

Despite their acknowledgement that commercial-in-confidence regulation might affect public interests, policy documents of medical licensing bodies and HTA committees such as EMA or NICE currently fail to specify:

(1) What measures for the protection of commercial interest remain available to manufacturers *after* market approval has been granted;

(2) Which types of public interests ought to be considered in evaluating the appropriateness of any measure designed to protect commercial interests post approval; and most importantly,

(3) What criteria guide the trade-off between public interest and legitimate measures for the protection of commercial interests.

This article briefly outlines the different definitions of commercial interests in the policies of EMA and NICE, with reference to a recent case analysis that described the attempt to access a specific set of (unpublished) clinical data from the EMA [6]. This article then specifies for seven stakeholder groups the public interest in full access to all trial data of pharmaceuticals and medical devices. Finally, the article argues that in light of the seven specifications of public interest in trial data access, there is a disproportionate focus on those measures that protect commercial interests (such as restricted access to trial data) inherent in current data protection policies.

What the article does not provide is a detailed guide to the multitude of respective policies that national and international agencies have drawn up with regard to trial data publication and protection of commercial interests, as such an analysis would certainly extend beyond the scope of one article. Furthermore, the article will not discuss the various reasons and past developments that underlie the current extent of secrecy in transparency policies of EMA and NICE (see e.g. [10,11]). We also refrain from seeking to evaluate which actor within the European health care sector would be most suitable to address any shortcomings that we outline, and we recognise that organisations like EMA are bound by European law, and their activities are regulated not by health policies, but complex European trade and commerce agreements.

### Definitions and specifications of commercial interests

It is noteworthy that despite broadly similar formulations of principles of confidentiality throughout the process of drug approval within the European Union, there is no definition that has been universally adopted by all member states. The EMA has therefore proposed that given the lack of such a common definition, commercial confidential information “shall mean any information which is not in the public domain or publicly available and where disclosure may undermine the economic interest of competitive position of the owner of the information” [7]. And NICE states that “[commercial] in confidence material [is] defined as confidential because its public disclosure could have an impact on the commercial interests of a particular company” [8].

Since the 1990s, different attempts have been made to encourage drug regulation agencies to decrease the extent of secrecy on unpublished clinical trial data [10,12]. While some reviews demonstrated that unpublished trials from drug-licensing applications added only a little to the data available from published trials [13], others showed how public disclosure of unpublished study results was critical to uncovering the evidence of harm [14] or the lack of effectiveness [15,16].

In a recent article, Gøtzsche and Jørgensen have chronicled the process of gaining access to unpublished trial data of two anti-obesity drugs from the EMA [6]. Their account gives two reasons provided by EMA for limiting access to trial data (as a measure to protect commercial interests): Unpublished trials held by the EMA may contain commercially sensitive information regarding (1) the clinical development programme of a new product, and (2) the most substantial part of the applicant's investment. The EMA argued that both types of information could be used by competitors to their economic advantage and that consequently, manufacturers applying for market approval had a vested (and justified) interest in their protection. In this case, no further commercial interests were alluded to. Whilst researching this article, we contacted the information services of three international organisations representing the research-based pharmaceutical industry to inquire if there were further reasons or relevant information to be considered beside those mentioned by the EMA, which would justify a restriction of access to data after market approval has been granted.^a^ After 4 weeks and a second inquiry, only one (the German *Association of Research-Based Pharmaceutical Companies*) responded and sent a recent law analysis paper on European licensing process. However, in this German language paper we did not find further reasons or information that justify a restriction of access to data after market approval has been granted [17].

Gøtzsche's and Jørgensen's account is interesting for another reason, as throughout the process of trying to gain access to trial data, neither the EMA (who for more than 2 years denied access) nor the European ombudsman who was involved as a mediator specifically addressed the question of potential overriding public interests that might be present in this case. In his assessment, the European ombudsman did not take a definitive stance on whether public interests were affected in this case, and restricted himself to pointing out that he was unable to see any commercial interest being undermined 'specifically and actually' by providing access to the relevant reports and protocols at the EMA [6]. This line of argument avoids a discussion of any potential public interests that might be affected. However, in order to assess the ethical justifiability of restriction of access to trial data more generally, it appears important to be more explicit about what types of public interest and their specifications are potentially at stake.

### Seven specifications of the public interest in full access to trial data

Decisions in clinical research, medical training, clinical care, public health, and health policy are all dependent on comprehensive and unbiased information on the potential benefits and harms of specific clinical or public health interventions. A major source of biased information on clinical or public health interventions is the restricted access to all existing data from clinical research. Biased information consequently leads to biased decisions that may violate the core principles of medical ethics; namely: beneficence, non-maleficence, respect of patient autonomy, and justice [18]. How these ethical principles are potentially violated will be explained in the following paragraphs, which specify the public interest in full access to trial data for seven different stakeholder groups. While the focus of this article is on the access to trial data *after* market approval has been granted, some of the seven stakeholder groups (e.g. research participants, clinical researchers, and research ethics committees) also have a strong interest in access to trial data *before* market approval has been granted (for reasons described below).

*Research participants* arguably have a strong interest in the release and public availability of the findings of any study in which they took part [19]. Participating in clinical trials carries health risks, which in spite of compensation can only be deemed ethically acceptable under certain conditions [20,21]. Paramount to these conditions is the generation of expected social benefits or value of such research. However, a biased publication of trial data, or a complete withholding thereof, appears to violate this basic principle. It should also be noted that it is questionable whether patients would actually continue to participate in research projects that carry certain risks when they are adequately informed that research findings that are helpful to inform health related decisions may be withheld because of commercial interests, even after market approval has been granted. To what extent this interest of research participants justifies unrestricted access to trial data must be judged with respect to the interests of the following stakeholders.

*Clinical researchers* planning new trials need data from earlier studies to formulate a sound research question, refine measurement instruments, and calculate adequate sample sizes [22]. Giving researchers access to all available data, thereby allowing them to specify research questions and to improve study designs, reduces the burden imposed upon study participants [23].

*Research ethics committees (REC)* and *institutional review boards (IRB)* have an interest in complete availability of trial data in order to determine the feasibility and all potential risks of clinical trials [23]. If the results of previous research projects are only partially or not at all available, this assessment of potential risks of new trials is potentially compromised, which in turn may put future trial participants at risk.

*Clinical practice guideline (CPG) development panels* and *health technology assessment (HTA) institutions* can judge best practice and develop reports and guidelines for treatment only based on publicly available information. Bias in published studies could potentially impact on assessments and thus on the actual guidelines that are being recommended [24]. A recent example is the case described by IQWiG, the German HTA institution, that uncovered unpublished trials that eventually led to a substantial revision of the risk-benefit assessment of the antidepressant reboxetin [5].

*Patients and their doctors* also draw on the information and recommendations from clinical practice guidelines and HTA reports during the process of shared clinical decision making. Biases in these guidelines and reports thus impact on the optimal, personalised course of treatment, and patients may be harmed unnecessarily [25].

*Public research sponsors* such as the British Wellcome Trust or the American National Institutes of Health (NIH) will want to know what the risks and expected benefits of a proposed project are, and what the added benefit of a study is. However, if previous unpublished research renders the proposed project redundant or of minor practical relevance, scarce public resources will be used inefficiently [26].

*Health insurance companies* (both statutory and private) heavily draw on the final reports of HTA and CPG groups when making coverage decisions. Biases in these reports thus impact on the sound and fair allocation of scarce financial resources [27].

## Discussion and conclusion

In order to comprehensively assess the impact of restricting access to trial data, and to reach ethically defensible decisions, it is crucial to take into account the seven specifications of public interest we have outlined above. Currently, this does not appear to be common practice: In the case recently described by Gøtzsche and Jørgensen, the EMA argued that it could not identify any overriding public interest in this particular case [6]. From an ethical perspective, however, it would have been useful (and appropriate) if EMA had argued *why* different public interests such as those outlined above did not affect the decision about data access in this specific case.

We would suggest that the tendency of medical licensing bodies to place the protection of commercial interest over the public interest in access to trial data lacks proportionality. This is particularly obvious in cases where restrictions to data access are upheld *after* a drug has already been approved and entered the market.

Of course, there might be strong commercial interest after market approval to withhold study findings that indicate (1) that the effects of specific drugs or medical devices are less positive and of lower clinical relevance than suggested by the published data, or (2) that the side effects are more severe and of greater clinical relevance than suggested by the published data. However, protecting commercial interests on these grounds does not appear to be justifiable on any ethical, practical or legal grounds. This leads to the conclusion that unless (1) there are other more legitimate reasons for continued restriction of access to trial data post market approval of a drug that have so far not been publicly discussed or (2) there are exceptional circumstances in which the public interest in trial data publication is negligible, the seven types of public interests discussed offer a rationale for a principle of full public disclosure of all trial data at the same time as market approval has been granted.

Licensing bodies such as the EMA as well as HTA institutions such as NICE should therefore provide further justification as to why commercial interests frequently override public interests in decisions to restrict data access. As argued above, the current decision-making practices of the EMA and NICE fail to account for different specifications of public interest. From an ethical perspective, it would therefore be appropriate for the EMA and NICE to revise their policies on data transparency: the default restriction to data should be changed to a default access to data. This also applies to other national institutions with similar transparency policies. Furthermore, the revised policies should specify (1) the reasons for protecting commercially sensitive trial data (before and after market access has been granted or denied) and (2) when these reasons override the seven specifications of public interest in informed decisions.

## Endnotes

^a^The organisations contacted were: *The International Federation of Pharmaceutical Manufacturers & Associations* (IFPMA), the *Pharmaceutical Research and Manufacturers of America* (PhRMA), and the German *Association of Research-Based Pharmaceutical Companies* (vfa).

## Competing interests

The authors declare that they have no competing interests.

## Authors’ contributions

DS wrote the first draft of the manuscript. JP added substantial content and DS finalized the manuscript. All authors read and approved the final manuscript.

## References

[B1] Dickersin K Rennie D Registering clinical trials JAMA 2003 290 4 516 523 10.1001/jama.290.4.516 12876095

[B2] De Angelis C Drazen JM Frizelle FA Haug C Hoey J Horton R Kotzin S Laine C Marusic A Overbeke AJ Clinical trial registration: a statement from the International Committee of Medical Journal Editors Ann Intern Med 2004 141 6 477 478 15355883 10.7326/0003-4819-141-6-200409210-00109

[B3] Turner EH Matthews AM Linardatos E Tell RA Rosenthal R Selective publication of antidepressant trials and its influence on apparent efficacy N Engl J Med 2008 358 3 252 260 10.1056/NEJMsa065779 18199864

[B4] Rising K Bacchetti P Bero L Reporting bias in drug trials submitted to the Food and Drug Administration: review of publication and presentation PLoS Med 2008 5 11 e217 discussion e217 10.1371/journal.pmed.0050217 19067477 PMC2586350

[B5] Wieseler B McGauran N Kaiser T Finding studies on reboxetine: a tale of hide and seek BMJ 2010 341 c4942 10.1136/bmj.c4942 20940211

[B6] Gotzsche PC Jorgensen AW Opening up data at the European Medicines Agency BMJ 2011 342 d2686 10.1136/bmj.d2686 21558364

[B7] EMA HMA/EMA recommendations on transparency November 2010) http://www.ema.europa.eu/docs/en_GB/document_library/Other/2010/12/WC500099536.pdf p. 2. In.; 2010

[B8] NICE Guide to the methods of technology appraisal June 2008) http://www.nice.org.uk/media/B52/A7/TAMethodsGuideUpdatedJune2008.pdf Appendix D, p. 72. In.; 2008

[B9] Eichler HG Abadie E Breckenridge A Leufkens H Rasi G Open clinical trial data for all? A view from regulators PLoS Med 2012 9 4 e1001202 10.1371/journal.pmed.1001202 22505851 PMC3323505

[B10] International Working Group on Transparency and Accountability in Drug Regulation Statement 1996 Health Action International,Dag Hammarskjöld Foundation, Amsterdam 10.3233/JRS-1996-9312 23510919

[B11] Lexchin J Transparency in Drug Regulation: Mirage or Oasis? In 2004 Canadian Centre for Policy Alternatives, Ottawa 10.1503/cmaj.1041446 PMC527339 15557590

[B12] Lexchin J Mintzes B Transparency in drug regulation: mirage or oasis? CMAJ : Canadian Medical Association journal = journal de l'Association medicale canadienne 2004 171 11 1363 1365 10.1503/cmaj.1041446 PMC527339 15557590

[B13] Hemminki E McPherson K Value of drug-licensing documents in studying the effect of postmenopausal hormone therapy on cardiovascular disease Lancet 2000 355 9203 566 569 10.1016/S0140-6736(99)03432-7 10683020

[B14] Nissen SE Wolski K Effect of rosiglitazone on the risk of myocardial infarction and death from cardiovascular causes The New England Journal of Medicine 2007 356 24 2457 2471 10.1056/NEJMoa072761 17517853

[B15] Jefferson T Jones M Doshi P Del Mar C Neuraminidase inhibitors for preventing and treating influenza in healthy adults: systematic review and meta-analysis BMJ 2009 339 b5106 10.1136/bmj.b5106 19995812 PMC2790574

[B16] Doshi P Neuraminidase inhibitors–the story behind the Cochrane review BMJ 2009 339 b5164 10.1136/bmj.b5164 19995813

[B17] Sträter B Europäische Rahmenbedingungen für den Schutz von geistigem Eigentum an Zulassungsunterlagen. Teil 1: Rahmenbedingungen für den Schutz des Zulassungsdossiers Pharm Ind 2011 73 8 1450 1458

[B18] Foundation ABIM ACP–ASIM Foundation, European Federation of Internal Medicine: Medical professionalism in the new millennium: a physicians' charter Lancet 2002 359 9305 520 522 11853819 10.1016/S0140-6736(02)07684-5

[B19] Delamothe T Whose data are they anyway? BMJ 1996 312 7041 1241 1242 10.1136/bmj.312.7041.1241 8634604 PMC2351079

[B20] CIOMS International Ethical Guidelines for Biomedical Research Involving Human Subjects 2002 Council for International Organizations of Medical Sciences, Geneva 14983848

[B21] WMA Declaration of Helsinki: Ethical Principles for Medical Research Involving Human Subjects 2008 World Medical Association, Seoul 19886379

[B22] Smith GD Increasing the accessibility of data BMJ 1994 308 6943 1519 1520 10.1136/bmj.308.6943.1519 8019302 PMC2540522

[B23] Levin LA Palmer JG Institutional review boards should require clinical trial registration Arch Intern Med 2007 167 15 1576 1580 10.1001/archinte.167.15.1576 17698679

[B24] Kopp IB Implications of publication bias on guideline development and appraisal Zeitschrift fur Evidenz, Fortbildung und Qualitat im Gesundheitswesen 2011 105 3 201 206 10.1016/j.zefq.2011.03.010 21530910

[B25] European Science Foundation Implementation of Medical Research in Clinical Practice 2011 European Science Foundation (ESF), Strasbourg

[B26] Caplan AL Will evidence ever be sufficient to resolve the challenge of cost containment? Journal of clinical oncology : official journal of the American Society of Clinical Oncology 2011 29 15 1946 1948 10.1200/JCO.2011.34.7039 21502551

[B27] WHO The world health report − Health systems financing: the path to universal coverage 2010 World Health Organization (WHO), Geneva 10.2471/BLT.10.078741 PMC2878164 20539847

